# TRIB1 regulates liver regeneration by antagonizing the NRF2-mediated antioxidant response

**DOI:** 10.1038/s41419-023-05896-9

**Published:** 2023-06-24

**Authors:** Xinyue Sun, Shuai Wang, Xiulian Miao, Sheng Zeng, Yan Guo, Anqi Zhou, Ying Chen, Yifei Chen, Fangqiao Lv, Zhiwen Fan, Yutong Wang, Yong Xu, Zilong Li

**Affiliations:** 1 State Key Laboratory of Natural Medicines, Department of Pharmacology, China Pharmaceutical University, Nanjing, China; 2 Department of General Surgery, Nanjing Drum Tower Hospital Affiliated with Nanjing University School of Medicine, Nanjing, China; 3 Institute of Biomedical Research, Liaocheng University, Liaocheng, China; 4 Stem Cell Center, Nanjing Drum Tower Hospital Affiliated with Nanjing University School of Medicine, Nanjing, China; 5 Department of Cell Biology, Municipal Laboratory for Liver Protection and Regulation of Regeneration, School of Basic Medical Sciences, Capital Medical University, Beijing, China; 6 Department of Pathology, Nanjing Drum Tower Hospital Affiliated with Nanjing University School of Medicine, Nanjing, China

**Keywords:** Liver diseases, Transcription, Cell biology

## Abstract

Robust regenerative response post liver injuries facilitates the architectural and functional recovery of the liver. Intrahepatic redox homeostasis plays a key role in liver regeneration. In the present study, we investigated the contributory role of Tribbles homolog 1 (Trib1), a pseudokinase, in liver regeneration and the underlying mechanism. We report that Trib1 expression was transiently down-regulated in animal and cell models of liver regeneration. Further analysis revealed that hepatocyte growth factor (HGF) repressed Trib1 transcription by evicting liver X receptor (LXRα) from the *Trib1* promoter. Knockdown of Trib1 enhanced whereas over-expression of Trib1 suppressed liver regeneration after partial hepatectomy in mice. Of interest, regulation of liver regenerative response by Trib1 coincided with alterations of intracellular ROS levels, GSH levels, and antioxidant genes. Transcriptional assays suggested that Trib1 influenced cellular redox status by attenuating nuclear factor erythroid 2-related factor 2 (Nrf2) activity. Mechanistically, Trib1 interacted with the C-terminus of Nrf2 thus masking a potential nuclear localization signal (NLS) and blocking nuclear accumulation of Nrf2. Finally, correlation between Trib1 expression, Nrf2 nuclear localization, and cell proliferation was identified in liver specimens taken from patients with acute liver failure. In conclusion, our data unveil a novel pathway that depicts Trib1 as a critical link between intracellular redox homeostasis and cell proliferation in liver regeneration.

## Introduction

The liver is the most important detoxification organ in the human body. When the wide array of injurious insults inflicted on the liver exceeds its compensatory capacity, the patients develop end-stage liver diseases (ESLD) and eventually succumb to liver failure. It is estimated that approximately two million people die from ESLD-related liver failure accounting for 3.5% of all deaths worldwide [1]. One of the compensatory functions the liver executes to combat injuries is its unique ability to regenerate [2]. Hepatic parenchymal cells, or hepatocytes, which constitute over 85% of the liver mass, exit the cell cycle and reside in a quiescent state following the completion of liver development. In response to a myriad of injurious, hepatocytes can be catapulted into cell cycling and start proliferating again to offset the loss of liver parenchyma in a process known as liver regeneration [3]. Hepatocyte growth factor (HGF), believed to emanate from sinusoidal endothelial cells, is the primary pro-proliferative stimulus to promote liver regeneration [4]. Full-fledged liver regeneration facilitates recovery from injuries and averts the incidents of liver failure. On the contrary, compromised liver regeneration precipitates the loss of key liver functions and contributes to the deterioration of liver failure [5].

Among the many factors that modulate liver regenerative response is the redox homeostasis in hepatocytes [6]. Nuclear factor erythroid 2-related factor 2 (Nrf2) is the master transcriptional regulator of antioxidative response [7]. Beyer et al. have shown that compared to the wild-type littermates, Nrf2 knockout (KO) mice exhibit delayed regenerative response following partial hepatectomy with a concomitant decrease in hepatic GSH levels likely attributable to the downregulation of antioxidant genes [8]. This observation has been further corroborated by Shirasaki et al. in a mouse model of portal vein branch ligation-induced liver injury [9]. In addition, HGF-induced proliferation of hepatocytes is synonymous with upregulation of Nrf2 expression and accelerated GSH synthesis [10, 11]. In contrast, deletion of the HGF receptor c-MET leads to prolonged elevation of ROS production and impaired proliferation of hepatocytes [12]. A basic leucine-zipper (bZip) transcription factor that recognizes and binds to the antioxidative response element (ARE) detected on the promoters of multiple genes encoding ROS-cleansing proteins, Nrf2 is retained in the cytoplasm and constrained in a dormant state by its inhibitor Kelch-like ECH associated protein 1 (Keap1); once liberated, Nrf2 trans-locates to the nucleus and activates antioxidative transcription [13]. How Nrf2 activity is modulated in the process of liver regeneration is not entirely understood.

Tribbles homolog 1 (Trib1) belongs to an evolutionarily conserved family of pseudokinases that play unique and diverse roles to regulate both physiological and pathological processes [14]. Tribbles, the founding member of this family, was identified in fruit flies (*Drosophila melanogaster*). Independent investigations by the Leptin laboratory [15], the Rørth laboratory [16], and the Wieschaus laboratory [17] unanimously demonstrated a pivotal role for Tribbles in cell proliferation. Specifically, Tribbles promote proteasomal degradation of two mitotic activators, String and Twine, to block cell cycling and allow mesoderm morphogenesis to proceed. Curiously, Trib1 appears to be able to support cell proliferation in human cancers [18–20]. In the present study, we designed experiments to address the following two questions: (1) what is the correlative and causal relationship between Trib1 and liver regeneration? and (2) whether Trib1 could alter intrahepatic redox status in the course of liver regeneration? Our data suggest that Trib1 expression is down-regulated during liver regeneration, which leads to Nrf2 nuclear accumulation and activation of antioxidants thereby promoting hepatocyte proliferation.

## Methods

### Animals

All animal experiments were reviewed and approved by the Nanjing University Ethics Committee on Laboratory Animals. Partial hepatectomy was performed as previously described [21, 22]. Acetaminophen (APAP) was dissolved in 1XPBS and the mice received a single injection at a dose of 300 mg/kg as previously described [23, 24]. Plasma LDH levels were measured using a commercially available kit (Abcam, ab102526) per vendor instructions.

### Cell culture, transient transfection, and reporter assay

Primary murine hepatocytes were isolated as previously described [25–27]. HEK293 cells were maintained in DMEM supplemented with 10% FBS as previously described [28, 29]. The Wnt3a cells (CRL2647, ATCC) were maintained in complete DMEM supplemented with 0.4 mg/ml G-418. Mouse recombinant HGF was purchased from R&D. The cells were treated with HGF (20 ng/ml) for 12–48 h as indicated. Trib1 promoter-luciferase construct was generated by amplifying genomic DNA spanning the proximal promoter and the first exon of Trib1 gene (-2000/+105) and ligating into a pGL3-basic vector (Promega). FLAG-tagged Trib1 and HA-tagged Nrf2 have been previously described [30, 31]. Mutagenesis was performed a QuikChange kit (Thermo Fisher Scientific, Waltham, MA, United States) as previously described [32, 33]. All DNA constructs were verified by direct sequencing. Small interfering RNAs were purchased from GenePharma. Cells were harvested 24-48 h after the transfection. Transient transfections were performed with Lipofectamine 2000. Luciferase activities were assayed using a luciferase reporter assay system (Promega) as previously described [34, 35].

### IPA analysis

Ingenuity pathway analysis (IPA) was performed using “Trib1” as keyword with the proprietary software developed by Qiagen (Hilden, Germany).

### Chromatin immunoprecipitation (ChIP)

Chromatin Immunoprecipitation (ChIP) assays were performed essentially as described before [36–39]. In brief, chromatin in control and treated cells were cross-linked with 1% formaldehyde. Cells were incubated in lysis buffer (150 mM NaCl, 25 mM Tris pH 7.5, 1% Triton X-100, 0.1% SDS, 0.5% deoxycholate) supplemented with protease inhibitor tablet and PMSF. DNA was fragmented into ~200 bp pieces using a Branson 250 sonicator. Aliquots of lysates containing 200 μg of protein were used for each immunoprecipitation reaction with anti-LXRα (Abcam, ab41902), anti-ELL2 (Proteintech, 12727-1), anti-MSC (Santa Cruz, sc293482), anti-KDM2A (Abcam, ab191387), anti-PRDM1 (Cell Signaling Tech, 9115), anti-Nrf2 (Cell Signaling Tech, 12721), or pre-immune IgG.

### Nrf2 activity assay

Nrf2 activity was measured by a kit purchased from Abcam (ab207223) as previously described [40]. Briefly, nuclear lysates were incubated with ARE oligos tethered to a reaction plate. Following the addition of an anti-Nrf2 primary antibody and an HRP-conjugated secondary antibody, binding of Nrf2 to the ARE oligos was detected by spectrometry at 450 nm.

### RNA isolation and real-time PCR

RNA was extracted with the RNeasy RNA isolation kit (Qiagen) as previously described [41, 42]. Reverse transcriptase reactions were performed using a SuperScript First-strand Synthesis System (Invitrogen) as previously described [43, 44]. Real-time PCR reactions were performed on an ABI Prism 7500 system with the following primers: *Ccna2*, 5′-TGGATGGCAGTTTTGAATCACC-3′ and 5′-CCCTAAGGTACGTGTGAATGTC-3′; *Ccne1*, 5′-CTCCGACCTTTCAGTCCGC-3′ and 5’-CACAGTCTTGTCAATCTTGGCA-3′; *Ccnb1*, 5′-CAATTATCGGAAGTGTCGGATCA-3′ and 5′-CTGGTGAACGACTGAACTCCC-3′; *Pcna*, 5′-TTTGAGGCACGCCTGATCC-3′ and 5′-GGAGACGTGAGACGAGTCCAT-3′; *Gclm*, 5′-AGGAGCTTCGGGACTGTATCC-3′ and 5′-GGGACATGGTGCATTCCAAAA-3′; *Nqo1*, 5′-AGGATGGGAGGTACTCGAATC-3′ and 5′-AGGCGTCCTTCCTTATATGCTA-3′; *Gsta1*, 5′-AAGCCCGTGCTTCACTACTTC-3′ and 5′-GGGCACTTGGTCAAACATCAAA-3′; *Gstp1*, 5′-ATGCCACCATACACCATTGTC-3′ and 5′-GGGAGCTGCCCATACAGAC-3′; *Gss*, 5′-5′-AAGCAGGCCATAGACAGGG-3′ and 5’AAAAGCGTGAATGGGGCATAC-3′. Ct values of target genes were normalized to the Ct values of housekeekping control gene (18s, 5′-CGCGGTTCTATTTTGTTGGT-3′ and 5′-TCGTCTTCGAAACTCCGACT-3′ for both human and mouse genes) using the DDCt method and expressed as relative mRNA expression levels compared to the control group which is arbitrarily set as 1.

### Protein extraction, immunoprecipitation, and western blotting

Whole-cell lysates were obtained by re-suspending cell pellets in RIPA buffer (50 mM Tris pH7.4, 150 mM NaCl, 1% Triton X-100) with freshly added protease inhibitor (Roche) as previously described [45, 46]. Nuclear proteins were prepared with the NE-PER Kit (Pierce) following the manufacturer’s recommendation. Specific antibodies or pre-immune IgGs (P.I.I.) were added to and incubated with cell lysates overnight before being absorbed by Protein A/G-plus Agarose beads (Santa Cruz). Precipitated immune complex was released by boiling with 1X SDS electrophoresis sample buffer. Alternatively, FLAG-conjugated beads (M2, Sigma) were added to and incubated with lysates overnight. The precipitated immune complex was eluted with 3X FLAG peptide (Sigma). For densitometrical quantification, densities of target proteins were normalized to those of β-actin as previously described [47, 48]. Data are expressed as relative protein levels compared to the control group which is arbitrarily set as 1.

### Histology

Histological analyses were performed essentially as described before [48–50]. Paraffin-embedded liver specimens were serially sectioned and were blocked with 10% normal goat serum for 1 h at room temperature and then incubated with anti-Ki67 (Abcam, ab15580, 1:200), anti-PCNA (Proteintech, 10205-1, 1:250), anti-Trib1 (Novus, NBP1-55386, 1:200), or anti-Nrf2 (Cell Signaling Tech, 12721, 1:200) antibodies. Staining was visualized by incubation with anti-rabbit secondary antibody and developed with a streptavidin-horseradish peroxidase kit (Pierce) for 20 min. Pictures were taken using an Olympus IX-70 microscope. Quantifications were performed with Image J.

### Human ALF specimens

Liver biopsies were collected from patients with ALF referring to Nanjing Drum Tower Hospital. Written informed consent was obtained from subjects or families of liver donors. All procedures that involved human samples were approved by the Ethics Committee of the Nanjing Drum Tower Hospital (approval reference #: 2020-155-01) and adhered to the principles outlined in the Declaration of Helsinki. Paraffin sections were stained with indicated antibodies. Slides were observed under a light microscope at high power (X40) by two pathologists independently in a double-blind fashion.

### Luminescence ROS assay

ROS levels were measured by the ROS-Glo system (Promega, G8820) as previously described [28, 29]. Briefly, a luminescence substrate solution was added to and incubated with cultured cells for 6 h followed by the addition of the dilution solution. Luminescence was measured using a microplate reader. Raw luminescence values were normalized by protein concentration and data were expressed as relative ROS levels compared to the control group.

### GSH quantification

Reduced glutathione (GSH) levels were measured with a commercially available kit (Abcam, ab235670) per vendor instructions.

### Immunofluorescence staining

The cells were fixed with 1% formaldehyde at room temperature and incubated with anti-Nrf2 (Cell Signaling Tech, 12721, 1:200) followed by incubation with donkey secondary antibodies (Jackson ImmunoResearch) as previously described [51, 52]. The nuclei were counterstained with DAPI (Sigma).

### Statistical analysis

One-way ANOVA with post-hoc Scheff´e analyses were performed by SPSS software (IBM SPSS v18.0, Chicago, IL, USA). Unless otherwise specified, values of *p* < 0.05 were considered statistically significant.

## Results

### Trib1 expression was down-regulated during the early phase of liver regeneration

**Fig. 1:**
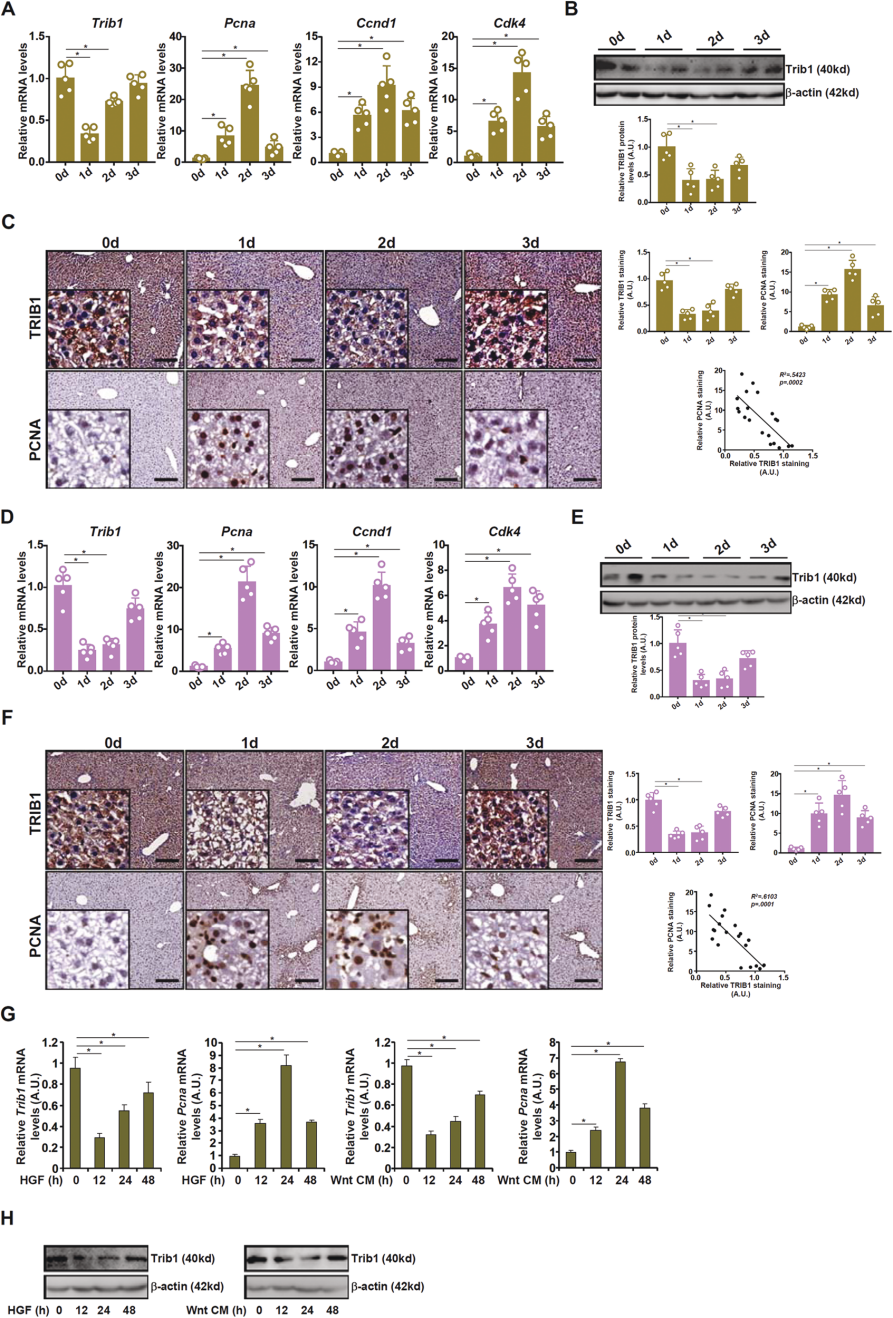
Trib1 expression was downregulated during the early phase of liver regeneration. **A**–**C** C57B6/L mice were subjected to partial hepatectomy (2/3 PHx) and sacrificed at indicated time points. Trib1 expression was examined by qPCR, western blotting, and immunohistochemical staining. *N* = 5 mice for each group. **D**–**F** C57B6/L mice were injected with acetaminophen (APAP, 300 mg/kg) and sacrificed at indicated time points. Trib1 expression was examined by qPCR, western blotting, and immunohistochemical staining. *N* = 5 mice for each group. **G**, **H** Primary murine hepatocytes were treated with HGF and harvested at indicated time points. Trib1 expression was examined by qPCR and western blotting. *N* = 3 biological replicates. Data are expressed as mean ± S.D. *p < 0.05, one-way ANOVA with post-hoc Scheff´e.

Trib1 expression was examined in several different models of liver regeneration. In the first animal model in which C57/B6 mice were subjected to partial hepatectomy (2/3 PHx), it was found that Trib1 expression was significantly down-regulated in the liver 24 h following the surgical procedure ahead of the up-regulation of proliferating cell nuclear antigen (*Pcna*), cyclin D1 (*Ccnd1*), and cyclin dependent kinase 4 (*Cdk4*), all of which peaked at 48 h after PHx as measured by quantitative PCR (Fig. 1A). Trib1 expression went up at 3d after the surgery, which coincided with the decline of PCNA expression (Fig. 1A). Western blotting (Fig. 1B) and immunohistochemical staining (Fig. 1C) confirmed that decrease of Trib1 expression accompanied the early phase of liver regeneration. Of note, there appeared to be an inverse correlation between Trib1 expression and PCNA expression in the regenerating livers (Fig. 1C). In the second model in which C57/B6 mice were injected with a single dose of acetaminophen (APAP) to induce acute liver failure, similar observations were made that Trib1 expression was down-regulated at 1d and 2d in the liver after APAP injection, which mirrored PCNA/cyclin D1/CDK4 up-regulation (Fig. 1D–F). Notably, there was a rapid upregulation of HGF expression the murine livers in both models of liver regeneration (Fig. S1).

Next, primary murine hepatocytes were treated with HGF or Wnt ligand (Wnt CM), both considered pro-regenerative, which triggered downregulation of Trib1 expression at mRNA (Fig. 1G) and protein (Fig. 1H) levels.

### HGF down-regulates Trib1 transcription via LXRα

**Fig. 2:**
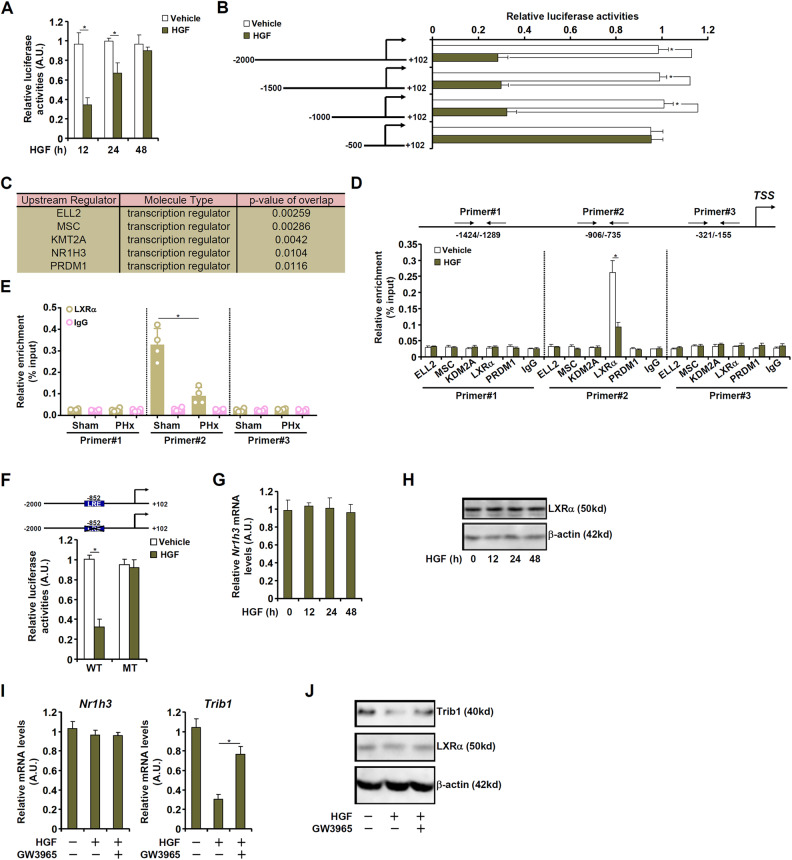
HGF downregulates Trib1 transcription via LXRα. **A** A *Trib1* promoter-luciferase construct was transfected into HepG2 cells followed by HGF (20 ng/ml) treatment and harvested at indicated time points. Luciferase activities were normalized by GFP fluorescence and protein concentration. *N* = 3 biological replicates. **B** *Trib1* promoter-luciferase constructs were transfected into HepG2 cells followed by HGF (20 ng/ml) treatment for 12 h. Luciferase activities were normalized by GFP fluorescence and protein concentration. *N* = 3 biological replicates. **C** IPA analysis of top upstream regulators for *Trib1*. **D** Primary murine hepatocyte was treated with HGF (20 ng/ml) for 12 h. ChIP assay was performed with indicated antibodies. *N* = 3 biological replicates. **E** C57B6/L mice were subjected to partial hepatectomy (2/3 PHx) and sacrificed 24 h after the procedure. ChIP assay was performed using liver lysates with anti-LXRα or IgG. *N* = 4 mice for each group. **F** Wild-type and mutant *Trib1* promoter-luciferase constructs were transfected into HepG2 cells followed by HGF (20 ng/ml) treatment for 12 h. Luciferase activities were normalized by GFP fluorescence and protein concentration. *N* = 3 biological replicates. **G**, **H** Primary murine hepatocytes were treated with HGF (20 ng/ml) and harvested at indicated time points. LXRα expression was examined by qPCR and western blotting. *N* = 3 biological replicates. **I**, **J** Primary murine hepatocytes were treated with HGF (20 ng/ml) and/or GW3965 (2μM). Trib1 expression was examined by qPCR and western blotting. *N* = 3 biological replicates. Data are expressed as mean ± S.D. *p < 0.05, one-way ANOVA with post-hoc Scheff´e.

The mechanism that contributes to Trib1 down-regulation during the early phase of liver regeneration was tackled by the following experiments. A Trib1 promoter-luciferase construct (−2000/+102) was transfected into HepG2 cells; HGF treatment elicit an early decrease of the Trib1 promoter activity suggesting that HGF could directly influence Trib1 transcription (Fig. 2A). Serial deletions were then introduced to the full-length Trib1 promoter to determine the HGF response region and it was discovered that whereas the −1000/+102 construct retained the responsiveness to HGF treatment the −500/+102 construct was non-responsive pointing to the existence of a potential HGF response element between −1000 and −500 (Fig. 2B). Next, several potential transcription factors were identified using the ingenuity pathway analysis (IPA) software (Fig. 2C), which include elongation factor for RNA Polymerase II 2 (ELL2), musculin (MSC), lysine methyltransferase 2A (KMT2A), nuclear receptor 1H3 (NR1H3, also known as liver X receptor α or LXRα), and PR domain zinc finger protein 1 (PRDM1). Chromatin immunoprecipitation (ChIP) assay showed that there was a strong association of LXRα, but not other transcription factors, with the Trib1 promoter between −1000 and −500 and that HGF treatment suppressed LXRα binding to the Trib1 promoter (Fig. 2D). Similarly, LXRα binding to the Trib1 promoter region was down-regulated in the regenerating livers compared to the quiescent livers following PHx (Fig. 2E). An LXR motif (AGGGTCA) was identified around −852 of the Trib1 promoter; mutation of this LXR motif abrogated trans-repression of the Trib1 promoter by HGF (Fig. 2F). Of note, HGF treatment did not appreciably influence LXRα expression at either mRNA (Fig. 2G) or protein (Fig. 2H) levels. LXRα expression was not altered in the liver following PHx or APAP injection (Fig. S1). The addition of a specific LXRα agonist (GW3965) blocked repression of Trib1 expression by HGF (Fig. 2I, J) suggesting that HGF might influence LXRα activity by modulating the bioavailability of endogenous LXRα ligands consistent with a previous report [53].

### Trib1 suppresses liver regeneration in vivo and in vitro

**Fig. 3:**
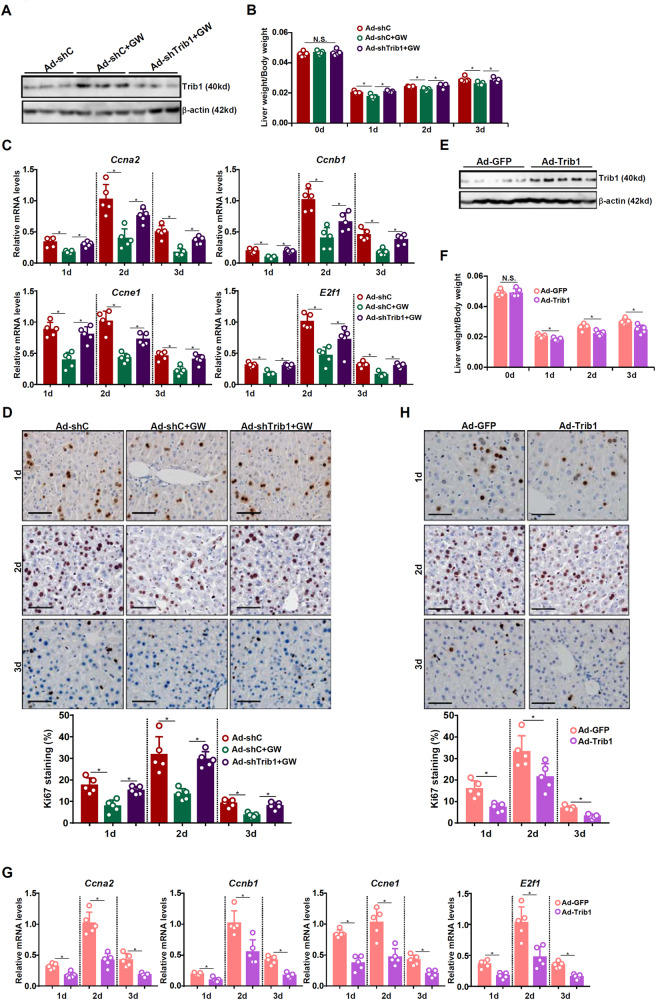
Trib1 suppresses liver regeneration in mice. **A**–**D** C57B6/L mice were injected with adenovirus carrying shRNA targeting Trib1 or an empty vector. GW3965 was administered by oral gavage 2 days prior to 2/3 partial hepatectomy. The mice were sacrificed at indicated time points after the surgery. **A** Trib1 expression levels were examined by western blotting. **B** Liver weight versus body weight. **C** Pro-proliferative gene expression levels were examined by qPCR. **D** Ki67 staining. *N* = 5 mice for each group. **E**–**H** C57B6/L mice were injected with adenovirus carrying a Trib1 vector or an empty vector prior to 2/3 partial hepatectomy. The mice were sacrificed at indicated time points after the surgery. **E** Trib1 expression levels were examined by western blotting. **F** Liver weight versus body weight. **G** Pro-proliferative gene expression levels were examined by qPCR. **H** Ki67 staining. *N* = 5 mice for each group. Data are expressed as mean ± S.D. *p < 0.05, one-way ANOVA with post-hoc Scheff´e.

Based on the observation that Trib1 expression was down-regulated during liver regeneration it was hypothesized that Trib1 might suppress liver regeneration. To test this hypothesis, C57/B6 mice were injected with adenovirus carrying shRNA targeting Trib1 (Ad-shTrib1) or a control vector (Ad-shC). Administration of the LXRα agonist GW3965 up-regulated Trib1 expression, which was blunted by Ad-shTrib1 injection (Fig. 3A). When the mice were subjected to 2/3 PHx and sacrifice at 1d, 2d, and 3d after the surgery it was found that GW3965 administration significantly delayed liver regeneration as assessed by liver weight versus body weight ratio (Fig. 3B), hepatic expression of pro-proliferative genes (Fig. 3C), and immunohistochemical staining of Ki67^+^ proliferating hepatocytes (Fig. 3D); the suppressive effect of GW3965 on liver regeneration was largely pre-empted by Trib1 knockdown. We also investigated whether or not Trib1 knockdown in and of itself could alter liver regeneration. As shown in Fig. S2, Trib1 depletion slightly but significantly accelerated liver regeneration following PHx as evidenced by liver weight/body weight ratios, expression levels of pro-regenerative genes, and Ki67 staining of proliferating hepatocytes.

In another set of experiments, C57/BL6 mice were injected with adenovirus carrying a Trib1 expression vector (Ad-Trib1) or a control vector (Ad-GFP) followed by partial hepatectomy (Fig. 3E). Trib1 over-expression was associated with decreased liver weight versus body weight ratio (Fig. 3F), downregulation of pro-proliferative genes in the liver (Fig. 3G), and fewer Ki67^+^ proliferating hepatocytes (Fig. 3H) all pointing to suppression of liver regenerative response.

In cultured primary murine hepatocytes, GW3965 treatment suppressed the expression of pro-proliferative genes and EdU incorporation into proliferating hepatocytes induced by HGF, both of which were attenuated by Trib1 knockdown (Fig. S4; Fig. S3 for knockdown efficiency). On the contrary, Trib1 over-expression was sufficient to halt HGF-induced proliferation of hepatocytes (Fig. S6; Fig. S5 for over-expression efficiency).

### Trib1 inhibits antioxidative response in vivo and in vitro

**Fig. 4:**
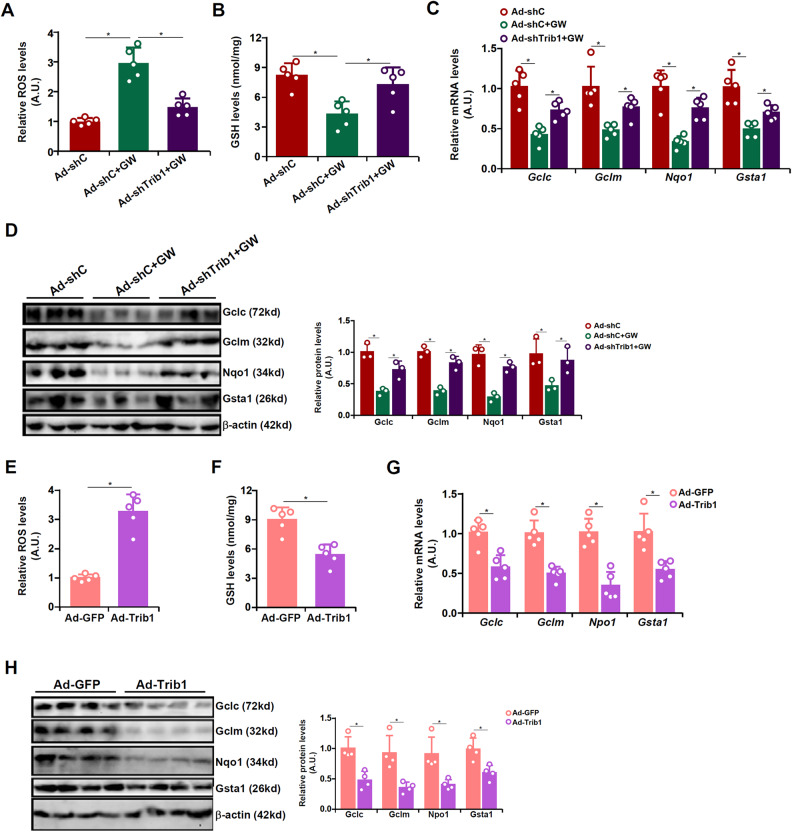
Trib1 inhibits antioxidative response in vivo. **A**–**D** C57B6/L mice were injected with adenovirus carrying shRNA targeting Trib1 or an empty vector. GW3965 was administered by oral gavage 2 days prior to 2/3 partial hepatectomy. The mice were sacrificed 48 h after the surgery. (A) Hepatic ROS levels were examined by a luminescence kit. **B** Hepatic GSH levels. Antioxidant gene expression levels were examined by qPCR (**C**) and western blotting (**D**). *N* = 5 mice for each group. **E**–**H** C57B6/L mice were injected with adenovirus carrying shRNA targeting Trib1 or an empty vector. The mice were sacrificed 48 h after the surgery. **E** Hepatic ROS levels were examined by a luminescence kit. **F** Hepatic GSH levels. Antioxidant gene expression levels were examined by qPCR (**G**) and western blotting (**H**). *N* = 5 mice for each group. Data are expressed as mean ± S.D. *p < 0.05, one-way ANOVA with post-hoc Scheff´e.

Intracellular redox status plays a key role modulating the liver regenerative response. We therefore asked the question as to whether the regulatory role of Trib1 in liver regeneration might be attributed to, at least in part, by the alterations of redox status in hepatocytes. Quantitative luminescence assay (Fig. 4A) and GSH quantification (Fig. 4B) suggested that GW3965 administration increased intrahepatic ROS levels with a simultaneous reduction in GSH levels, both of which were mollified by Trib1 knockdown. These changes in redox status following GW3965 administration coincided with down-regulation of antioxidant genes including glutamate-cysteine ligase catalytic subunit (*Gclc*), glutamate-cysteine ligase modifier subunit (*Gclm*), NADPH quinone dehydrogenase 1 (*Nqo1*), and glutathione S-transferase alpha 1 (*Gsta1*), a trend which was partially reversed by Trib1 knockdown (Fig. 4C, D). On the contrary, Trib1 over-expression increased ROS levels, decreased GSH levels, and down-regulated antioxidant genes in the regenerating livers (Fig. 4E–H). In cultured primary hepatocytes, Trib1 knockdown ameliorated the effects of GW3965 treatment on ROS levels, GSH levels, and expression levels of antioxidant genes whereas Trib1 over-expression suppressed HGF-induced ROS reduction, GSH production, and induction of antioxidant genes (Fig. S7). Together, these data suggest that Trib1 might be a link between intracellular redox status and cell proliferation in hepatocytes.

### Trib1 suppresses Nrf2 activity during liver regeneration

**Fig. 5:**
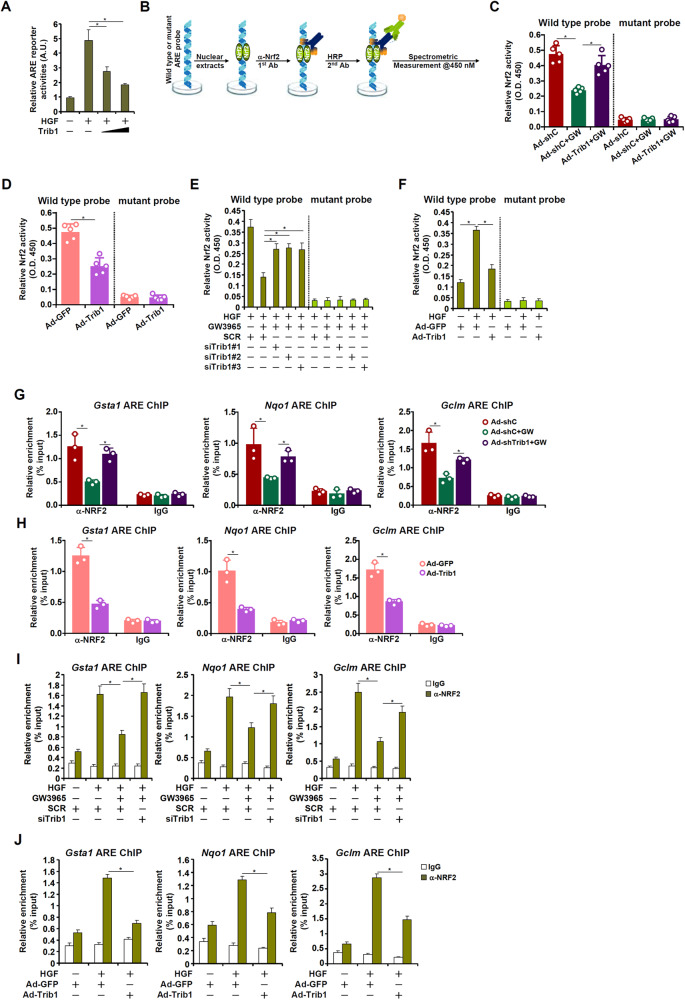
Trib1 suppresses Nrf2 activity during liver regeneration. **A** An ARE reporter was transfected into HepG2 cells with or without increasing doses of Trib1 followed by treatment with HGF (20 ng/ml). Luciferase activities were normalized by protein concentration and GFP fluorescence. *N* = 3 biological replicates. **B** A schematic diagram of the Nrf2 activity assay. **C** C57B6/L mice were injected with adenovirus carrying shRNA targeting Trib1 or an empty vector. GW3965 was administered by oral gavage 2 days prior to 2/3 partial hepatectomy. The mice were sacrificed 48 h after the surgery and liver tissues were assayed for Nrf2 activity. *N* = 5 mice for each group. **D** C57B6/L mice were injected with adenovirus carrying a Trib1 vector or an empty vector prior to 2/3 partial hepatectomy. The mice were sacrificed 48 h after the surgery and liver tissues were assayed for Nrf2 activity. *N* = 5 mice for each group. **E** Primary murine hepatocytes were transfected with siRNA targeting Trib1 or scrambled siRNA (SCR) followed by treatment with HGF (20 ng/ml) and/or GW3965 (2 μM) for 24 h. Nrf2 activity was assayed as described in Methods. *N* = 3 biological replicates. **F** Primary murine hepatocytes were transduced with indicated adenovirus followed by treatment with HGF (20 ng/ml) for 24 h. Nrf2 activity was assayed as described in “Methods”. *N* = 3 biological replicates. **G** C57B6/L mice were injected with adenovirus carrying shRNA targeting Trib1 or an empty vector. GW3965 was administered by oral gavage 2 days prior to 2/3 partial hepatectomy. The mice were sacrificed 48 h after the surgery and ChIP assay was performed with anti-Nrf2 or IgG using liver tissues. *N* = 3 mice for each group. **H** C57B6/L mice were injected with adenovirus carrying a Trib1 vector or an empty vector prior to 2/3 partial hepatectomy. The mice were sacrificed 48 h and ChIP assay was performed with anti-Nrf2 or IgG using liver tissues. *N* = 3 mice for each group. **I** Primary murine hepatocytes were transfected with indicated siRNAs followed by treatment with HGF (20 ng/ml) and GW3965 (2 μM) for 24 h. ChIP assay was performed with anti-Nrf2 or IgG. *N* = 3 biological replicates. **J** Primary murine hepatocytes were transduced with indicated adenovirus followed by treatment with HGF (20 ng/ml) for 24 h. ChIP assay was performed with anti-Nrf2 or IgG. *N* = 3 biological replicates. Data are expressed as mean ± S.D. *p < 0.05, one-way ANOVA with post-hoc Scheff´e.

Nrf2 is a master regulator of intracellular redox status by programming transcription of antioxidant genes. We tackled the question as to whether Trib1 could influence Nrf2 activity in the context of liver regeneration. First, it was observed that HGF treatment strongly stimulated the activity of a reporter driven by the Nrf2 binding motif (ARE) whereas Trib1 over-expression dose-dependently repressed the ARE reporter in the hepatocytes (Fig. 5A). Next, a spectrometric assay was used to determine nuclear Nrf2 activity (Fig. 5B). It was found that Trib1 knockdown blunted the negative effects of GW3965 treatment on Nrf2 activity both in liver tissues (Fig. 5C) and in primary hepatocytes (Fig. 5E). In contrast, Trib1 over-expression blocked the stimulation of Nrf2 activity by HGF treatment (Fig. 5D, F). Finally, chromatin immunoprecipitation (ChIP) confirmed that GW3965 interfered with the recruitment of Nrf2 to its target promoters, which was pre-empted by Nrf2 knockdown (Fig. 5G, I). Trib1 over-expression, on the other hand, blocked Nrf2 from binding to its target promoters (Fig. 5H, J). Typically, there is a second regeneration peak (4d-6d) following PHx representing the second round of hepatocyte mitosis. Of note, no significant changes were detected in Trib1 expression, Nrf2 expression, hepatic ROS production, or plasma LDH levels at 4d, 5d, and 6d post-PHx compared to 0d (Fig. S8). These data combined suggest that Trib1 might be a de novo inhibitor of Nrf2 activity in hepatocytes.

### Trib1 interacts with Nrf2 to block its nuclear translocation

**Fig. 6:**
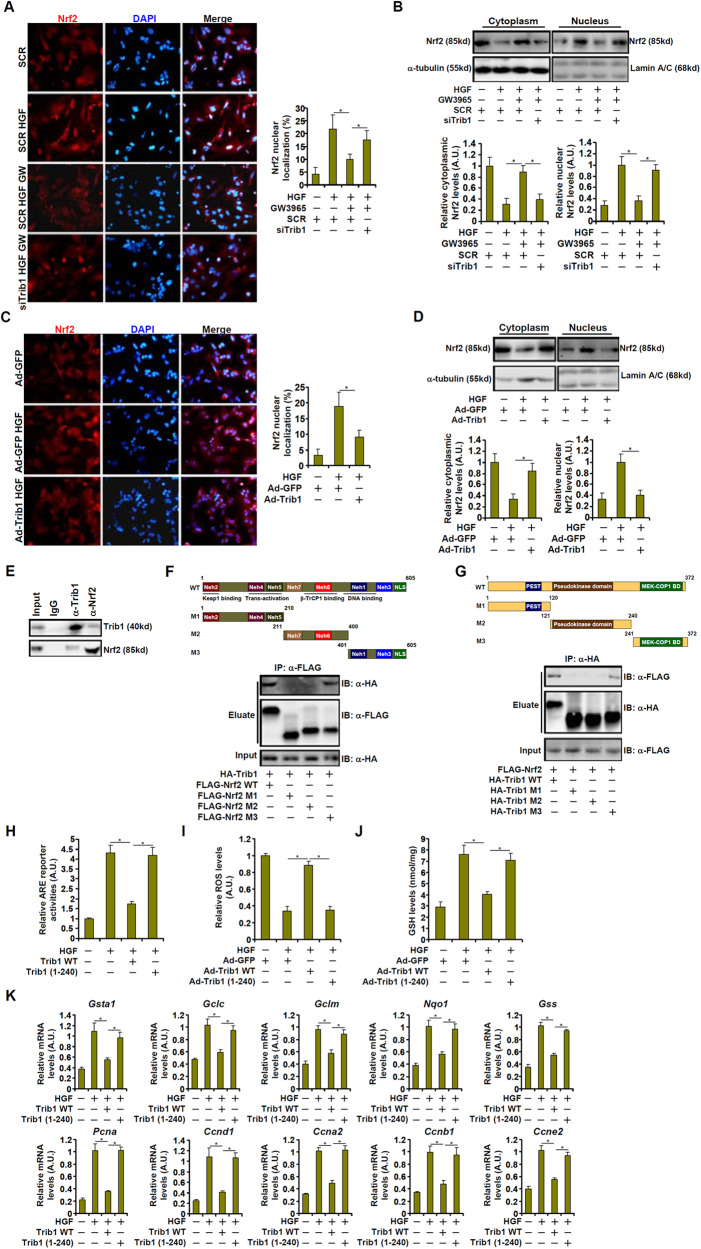
Trib1 interacts with Nrf2 to block its nuclear translocation. **A** Primary murine hepatocytes were transfected with indicated siRNAs followed by treatment with HGF (20 ng/ml) and GW3965 (2 μM) for 24 h. Immunofluorescence staining was performed with anti-Nrf2. **B** Primary murine hepatocytes were transfected with indicated siRNAs followed by treatment with HGF (20 ng/ml) and GW3965 (2 μM) for 24 h. Nrf2 in cytoplasmic and nuclear fractions was examined by western blotting. **C** Primary murine hepatocytes were transduced with indicated adenovirus followed by treatment with HGF (20 ng/ml) for 24 h. Immunofluorescence staining was performed with anti-Nrf2. **D** Primary murine hepatocytes were transduced with indicated adenovirus followed by treatment with HGF (20 ng/ml) for 24 h. Nrf2 in cytoplasmic and nuclear fractions was examined by western blotting. **E** Immunoprecipitation was performed with anti-Trib1, anti-Nrf2, or IgG using lysates extracted from primary murine hepatocytes. **F** HEK293 cells were transfected with FLAG-Trib1 and HA-tagged Nrf2. Immunoprecipitation was performed with anti-FLAG. **G** HEK293 cells were transfected with FLAG-Trib1 and HA-tagged Nrf2. Immunoprecipitation was performed with anti-HA. (**H**) An ARE reporter was transfected into HepG2 cells with wild-type or truncated Trib1 followed by treatment with HGF (20 ng/ml) for 24 h. Luciferase activities were normalized by protein concentration and GFP fluorescence. **I**–**K** Primary murine hepatocytes were transduced with indicated adenovirus followed by treatment with HGF (20 ng/ml) for 24 h. **I** ROS levels. **J** Hepatic GSH levels. **K** Gene expression levels were examined by qPCR. *N* = 3 biological replicates. Data are expressed as mean ± S.D. *p < 0.05, one-way ANOVA with post-hoc Scheff´e.

Altered Nrf2 transcriptional activity could stem from its expression and/or nuclear enrichment. Quantitative PCR and western blotting showed that neither Trib1 over-expression nor its knockdown significantly impacted Nrf2 expression in vivo (Fig. S9) and in vitro (Fig. S10). Nrf2 expression was not altered in the liver following PHx or APAP injection (Fig. S1). Nrf2 Immunofluorescence staining (Fig. 6A) and cell fractionation/western blotting (Fig. 6B) showed that HGF treatment stimulated Nrf2 translocation to the nucleus in hepatocytes; GW3965 treatment suppressed nuclear translocation of Nrf2, which was relieved by Trib1 knockdown. In addition, Nrf2 over-expression directly blocked HGF-induced nuclear migration of Nrf2 (Fig. 6C, D).

Because immunoprecipitation assay suggested that Trib1 and Nrf2 interacted with each in hepatocytes (Fig. 6E), we sought to delineate the domain(s) within Trib1 and Nrf2 that could mediate the interaction. To this end, truncation mutants for Nrf2 (Fig. 6F) and Trib1 (Fig. 6G) were generated and tested in co-IP assays. Nrf2 contains an N-terminal domain that mediates its interaction with Keap1 and transcriptional regulation of target genes, a central domain that can be targeted by β-TrCP1 for proteasomal degradation, and a C-terminal domain that controls its binding to DNA; the nuclear localization signal (NLS) is within the C-terminus (Fig. 6F). Co-IP results suggested that the C-terminal domain containing the NLS, but neither the N-terminal domain nor the central domain, was able to interact with Trib1 (Fig. 6F). On the other hand, Trib1 protein can be divided into three parts, an N-terminal domain that modulates its turnover (PEST), a central pseudokinase domain, and a C-terminal domain that serves a docking site for MEK and COP1 (Fig. 6G). Co-IP results suggested that the C-terminus of Trib1 was sufficient for its interaction with Nrf2 (Fig. 6G).

To further verify that the C-terminal domain of Trib1 was essential for brokering the Trib1-Nrf2 interaction and consequently sequestering Nrf2 in the cytoplasm to influence intracellular redox status and cell proliferation, we compared the effects of wild-type Trib1 and a mutant Trib1 harboring C-terminal truncations (1-240). The mutated Trib1 no longer interacted with Nrf2 (Fig. S11) and lost the ability to block Nrf2 nuclear accumulation when over-expressed (Fig. S12). As a result, Trib1 (1-240) over-expression failed to affect Nrf2 activity (Fig. 6H), was unable to alter cellular ROS levels (Fig. 6I, J), and left the expression levels of antioxidant and pro-proliferation genes intact (Fig. 6K).

### Correlation between TRIB1 expression, NRF2 nuclear localization, and hepatocyte proliferation in human ALF specimens

**Fig. 7:**
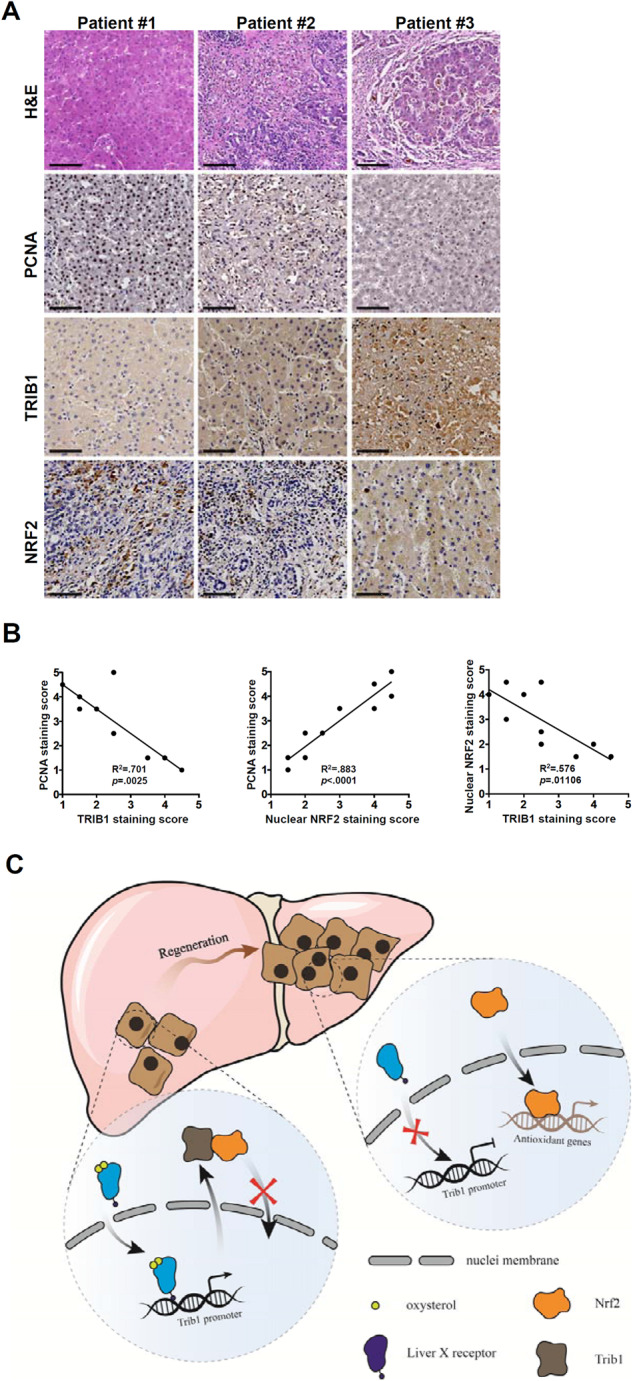
Correlation between TRIB1 expression, NRF2 nuclear localization, and hepatocyte proliferation in human ALF specimens. **A** Human liver biopsy specimens were stained with anti-TRIB1, anti-NRF2, and anti-PCNA. **B** Linear regression was performed with Graphpad. *N* = 9. **C** A schematic model. In quiescent hepatocytes, LXRα binds to the Trib1 promoter and activates Trib1 transcription. Trib1 interacts with Nrf2 to sequester Nrf2 in the cytoplasm thus turning off the transcription of antioxidant genes. In proliferating hepatocytes during live regeneration, deactivation of LXRα shuts down Trib1 transcription, which unleashes Nrf2 from sequestration. Nrf2 translocates to the nucleus and activates the transcription of antioxidant genes to modulate intracellular redox status fueling cell proliferation.

Finally, we sought to validate our model in humans using biopsy specimens from patients with acute liver failure. Immunohistochemical staining revealed that samples with strong proliferation of hepatocytes (PCNA) coincided with low Trib1 expression and prominent Nrf2 nuclear accumulation. In contrast, samples with weak proliferation of hepatocytes exhibited high Trib1 expression with Nrf2 mostly in the cytoplasm (Fig. 7A). Linear regression confirmed these observations to support the model that an inverse correlation existed between Trib1 expression, Nrf2 nuclear localization, and hepatocyte proliferation (Fig. 7B).

## Discussion

Liver regeneration is acutely influenced by intrahepatic redox status [6]. Nrf2-mediated regulation of antioxidative response contributes to hepatocyte proliferation and liver regeneration [8]. Here we describe a novel layer of mechanism in this process where LXRα-regulated Trib1 suppresses liver regeneration via Nrf2 sequestration to modulate redox homeostasis in hepatocytes (Fig. 7C).

We show here that LXRα directly binds to the Trib1 promoter and activates Trib1 transcription in hepatocytes. LXRα is known to exert anti-inflammatory effects in a wide range of pathophysiological processes and considered to be therapeutically attractive in coronary heart disease and Alzheimer’s disease [54, 55]. However, LXRα agonists have been reported to instigate severe hepatic adversity in rodents, non-human primates, and humans [56, 57]. Lo Sasso et al. observed that in C57/B6 mice subjected to partial hepatectomy, activation of LXRα by GW3965 was associated with impaired hepatocyte proliferation and delayed liver regeneration [53]. On the contrary, Zhang et al. suggested that blockade of LXRα activation restored liver regeneration in mice fed on a high-fat diet [58]. It is generally believed that liver regeneration can be fueled by glucose supplementation and suppression of hepatic lipogenesis [59]. Of note, hepatic over-expression of Trib1 leads to reduced lipid accumulation but increased glucose output [60, 61], suggesting that regulation of liver regeneration by Trib1 is unlikely attributable to LXRα-mediated metabolic reprogramming. Instead, multiple studies have pointed to an inverse correlation between LXRα and NRF2. NRF2 competes with FXR to deprive LXRα of a dimerization partner thereby leading to deactivation of LXRα target genes [62]. LXRα, on the other hand, represses NRF2 activity by acting as a heterodimer with RXRα [63]. In light of our data, we propose that Trib1 regulates liver regeneration through bridging the LXRα-NRF2 antagonism.

Our data suggest that Trib1 contributes to liver regeneration by regulating ROS levels. This observation is consistent with several previous reports that implicate the Tribbles family of proteins in cellular redox homeostasis although the underlying mechanisms appear to be divergent. Ito et al. have reported that Trib1 promotes ROS production in myeloid leukemia cells by cooperating with the E3 ligase COP1 to target acetyl-CoA carboxylase 1 (ACC1) for proteasomal degradation [64]. Trib1 is also able to promote macrophage polarization by stimulating ROS production likely via the JAK-STAT signaling pathway [65]. Wang and colleagues have shown that Trib2, unlike Trib1, curbs ROS production by modulating the activity of ubiquitin E3 ligases and protecting glutathione peroxidase 4 (GPX4), a major antioxidant, from proteasomal degradation [66, 67]. Still, there are studies to suggest that Trib3 safeguards cells from oxidative stress-induced apoptosis by acting as a repressor of the transcription factor ATF4 to down-regulate ROS production [68, 69]. In accordance, Wu et al. have recently reported that NIPI-3, a Trib3 ortholog found in *C. elegans*, represses the transcription of Vhp-1, a dual-specificity phosphatase homologous to mammalian DUSP8, by targeting C/EBP to degradation; reduced Vhp-1 expression elevates phosphorylation levels of SKN-1 (Nrf in *C. elegans*) and stimulates its activity [70]. Altered ROS production is often considered as a readout of mitochondrial stress. Indeed, there is evidence to show that Trib1 expression is sensitive to and may contribute to mitochondrial dysfunction. For instance, Soubeyrand et al. showed that treatment of hepatoma cells (HepG2) with oligomycin, a disruptor of mitochondrial function, led to rapid and sustained elevation of Trib1 expression [71]. Interestingly, down-regulation of Trib1 expression has been detected in skeletal muscle tissues with increased mitochondria biogenesis in human subjects following exercising [72]. Therefore, our finding that links Trib1-modulated Nrf2 to skewed redox status in proliferating hepatocytes further cements and expands the role Tribbles proteins play in mitochondrial biology.

We show here that Trib1 interacts directly with Nrf2 via its C-terminal MEK binding domain. However, other possibilities via which Trib1 may regulate Nrf2 activity cannot be excluded. First, Trib1, similar to other Tribbles proteins, primarily functions a molecular scaffold to mediate signaling transduction. In this context, it should be noted that binding of MEK1 (MKK1) to Trib1 leads to differential regulation of MAPK signaling with a selective inhibition of p38 activity [73]. A study published by Sun et al. indicates that phosphorylation of Nrf2 by p38 at five distinct serine/threonine residues, without impacting the Nrf2-Keap1 interaction, moderately but significantly boosts Nrf2 activity likely by promoting Nrf2 nuclear translocation [74]. Thus, it is tempting to speculate that Trib1 may suppress Nrf2 activity by negatively regulating p38 signaling. Second, our data indicate that Trib1 interacts with the C-terminal DNA-binding domain of Nrf2 (Fig. 6F). Joo et al. have reported that phosphorylation of serine residue 550 within the C-terminal DNA binding domain of Nrf2 serves as a prerequisite for Nrf2 nuclear trans-location [75]. It is likely that Trib1 may compete with AMPK for Nrf2 binding and block AMPK-dependent Nrf2 phosphorylation thereby leading to Nrf2 retention in the cytoplasm. Third, previous studies have indicated that Trib1 can be subjected to post-translational modifications. The C-terminal domain of Trib1 that mediates its interaction with Nrf2 can be subjected to phosphorylation by p38 [76]. Because HGF induces pro-regenerative responses partly through p38 signaling [77], it is possible that p38 activation downstream of HGF may phosphorylate Trib1 to alter the interface and weaken the Trib1-Nrf2 interaction to release Nrf2 from sequestration. These lingering issues certainly deserve further investigation.

One key issue left unaddressed by the present study is whether zonation of Trib1 plays a role in liver regeneration. It is widely accepted that spatial arrangements of different molecules cater to distinct metabolic requirements posted by different cell lineages during liver regeneration [78]. A seminal paper by the Corlu laboratory reveals that glutathione transferases P1/P2, prototypical Nrf2 targets, are exclusively located to the periportal intra-lobular region of the liver where nascent hepatocytes originate during liver regeneration [79]. Indeed, Rada et al. [80] and Skoko et al. [81] have provided corroborating evidence that Nrf2-mediated antioxidative response is emphatically compartmentalized owing to Nrf2 zonation, which may be critical for maintaining liver homeostasis and for disease pathogenesis. Because our data suggest that Trib1 contributes to liver regeneration by modulating Nrf2 activity, it would be of great interest to determine in future studies whether or not Trib1 displays Nrf2-like zonation in the liver.

Despite the advances offered by our findings, several questions remain unanswered. First, Trib1 has been well characterized as a proto-oncogene that stimulates proliferation of leukemia cells [82]. It is currently unknown how Trib1 can differentiate between physiological (e.g., regeneration) and pathological (e.g., cancer) cell proliferation. Second, Trib1 is ubiquitously expressed in both parenchymal cells and non-parenchymal cells (NPCs, e.g., macrophages and sinusoidal endothelial cells) in the liver. Whether and, if so, how Trib1 in NPCs contributes to liver regeneration awaits further investigation. Third, ROS-independent mechanisms whereby Trib1 regulates liver regeneration have not been explored in this study. Finally, although an inverse correlation between Trib1 expression and liver regeneration was identified in patients with ALF (Fig. 7), how to exploit this observation to devise interventional strategy awaits further studies.

In summary, our data provide novel mechanistic insights for liver regeneration. Future investigations should take advantage of multi-omics based strategies to translate these findings and devise therapeutic regimens to treat liver failure.

## Supplementary information

online supplementary material

checklist

## Data Availability

The data that support the findings of this study are available upon reasonable request.
