# Supplementary material for: TRIB1 regulates liver regeneration by antagonizing the NRF2-mediated antioxidant response
Source: Cell Death Dis. 2023 Jun 24;14(6):372. doi: 10.1038/s41419-023-05896-9 (PMC10290656; doi:10.1038/s41419-023-05896-9)
Supplement: Supplementary file 1 — online supplementary material [file 41419_2023_5896_MOESM1_ESM.doc]

**Sun XY et al: Trib1 suppresses liver regeneration by antagonizing Nrf2-mediated antioxidative response**

**Online supplementary material**

**Supplementary figures: 12**

**Supplementary table: 1**

**Fig.S1:** (**A**) C57B6/L mice were subjected to partial hepatectomy (2/3 PHx) and sacrificed at indicated time points. Gene expression levels were examined by qPCR. N=5 mice for each group. (**B**) C57B6/L mice were injected with acetaminophen (APAP, 300mg/kg) and sacrificed at indicated time points. Gene expression levels were examined by qPCR. N=5 mice for each group.

**Fig.S2:** C57B6/L mice were injected with adenovirus carrying shRNA targeting Trib1 or an empty vector followed by 2/3 partial hepatectomy. The mice were sacrificed at indicated time points after the surgery. (**A**) Liver weight versus body weight. (**B**) Pro-proliferative gene expression levels were examined by qPCR. (**C**) Ki67 staining. N=5 mice for each group.

**Fig.S3:** (**A, B**) Primary murine hepatocytes were transfected with indicated siRNAs. The cells were harvested 48h after transfection and Trib1 expression was examined by qPCR and Western.

**Fig.S4:** (**A, B**) Primary murine hepatocytes were transfected with siRNA targeting Trib1 or scrambled siRNA followed by treatment with HGF (20ng/ml) and GW3965 (2μM) for 24h. Pro-proliferative gene expression levels were examined by qPCR. Cell proliferation was evaluated by EdU incorporation.

**Fig.S5:** (**A, B**) Primary murine hepatocytes were transduced with indicated adenovirus. The cells were harvested 48h after transduction and Trib1 expression was examined by qPCR and Western.

**Fig.S6:** (**A, B**) Primary murine hepatocytes were transduced with indicated adenovirus followed by treatment with HGF (20ng/ml) for 24h. Pro-proliferative gene expression levels were examined by qPCR. Cell proliferation was evaluated by EdU incorporation.

**Fig.S7:** (**A-C**) Primary murine hepatocytes were transfected with siRNA targeting Trib1 or scrambled siRNA followed by treatment with HGF (20ng/ml) and GW3965 (2μM) for 24h. (A) ROS levels were examined by a luminescence kit. (B) GSH levels. Antioxidant gene expression levels were examined by qPCR (C). (**D-F**) Primary murine hepatocytes were adenovirus carrying Trib1 or an empty followed treatment with HGF. (D) ROS levels were examined by a luminescence kit. (E) GSH levels. Antioxidant gene expression levels were examined by qPCR (F).

**Fig.S8:** C57B6/L mice were subjected to partial hepatectomy (2/3 PHx) and sacrificed at indicated time points. (**A**) Trib1 expression was examined by qPCR. (**B**) Nrf2 expression was examined by qPCR. (**C**) Hepatic ROS levels were examined by a luminescence kit. (**D**) Plasma LDH levels.

**Fig.S9:** (**A**) C57B6/L mice were injected with adenovirus carrying shRNA targeting Trib1 or an empty vector. GW3965 was administered by oral gavage 2 days prior to 2/3 partial hepatectomy. The mice were sacrificed 48h after the surgery. Nrf2 expression was examined by qPCR. (**B**) C57B6/L mice were injected with adenovirus carrying shRNA targeting Trib1 or an empty vector. The mice were sacrificed 48h after the surgery. Nrf2 expression was examined by qPCR.

**Fig.S10:** (**A**) Primary murine hepatocytes were transfected with siRNA targeting Trib1 or scrambled siRNA followed by treatment with HGF (20ng/ml) and GW3965 (2μM) for 24h. Nrf2 expression was examined by qPCR. (**B**) Primary murine hepatocytes were adenovirus carrying Trib1 or an empty followed treatment with HGF. Nrf2 expression was examined by qPCR.

**Fig.S11:** HEK293 cells were transfected with FLAG-Trib1 and HA-tagged Nrf2. The cells were harvested 48h after transfection and immunoprecipitation was performed with anti-HA.

**Fig.S12:** Primary murine hepatocytes were transduced with indicated adenovirus followed by treatment with HGF (20ng/ml) for 24h. Nrf2 in cytoplasmic and nuclear fractions was examined by Western blotting.

**Supplementary Table I: ALF patient vitals**

| **Patient**  **ID** | **Gender** | **Age**  **(y)** | **Temp**  **(^o^C)** | **BP**  **(mmHg)** | **ALT**  **(U/dL)** | **AST**  **(U/dL)** | **LDH**  **(U/dL)** |
| --- | --- | --- | --- | --- | --- | --- | --- |
| 1 | M | 42 | 36.8 | 103/58 | 1726 | 1124 | 2815 |
| 2 | M | 29 | 36.4 | 123/84 | 729 | 175 | 346 |
| 3 | F | 30 | 36.8 | 102/71 | 547 | 548 | 890 |
| 4 | M | 29 | 36.9 | 118/76 | 1687 | 633 | 272 |
| 5 | M | 26 | 36.3 | 110/70 | 583 | 1791 | 1684 |
| 6 | M | 63 | 36.2 | 145/75 | 1598 | 1578 | 8870 |
| 7 | M | 44 | 36.0 | 132/79 | 587 | 728 | 913 |
| 8 | F | 44 | 37.0 | 122/78 | 1525 | 1460 | 881 |
| 9 | M | 37 | 36.6 | 118/77 | 909 | 156 | 781 |
